# Heat Waves in the United States: Mortality Risk during Heat Waves and Effect Modification by Heat Wave Characteristics in 43 U.S. Communities

**DOI:** 10.1289/ehp.1002313

**Published:** 2010-11-18

**Authors:** G. Brooke Anderson, Michelle L. Bell

**Affiliations:** 1 Environmental Engineering Program and; 2 School of Forestry and Environmental Studies, Yale University, New Haven, Connecticut, USA

**Keywords:** climate change, extreme temperature events, heat waves, human health, mortality, temperature-mortality relationships

## Abstract

**Background:**

Devastating health effects from recent heat waves, and projected increases in frequency, duration, and severity of heat waves from climate change, highlight the importance of understanding health consequences of heat waves.

**Objectives:**

We analyzed mortality risk for heat waves in 43 U.S. cities (1987–2005) and investigated how effects relate to heat waves’ intensity, duration, or timing in season.

**Methods:**

Heat waves were defined as ≥ 2 days with temperature ≥ 95th percentile for the community for 1 May through 30 September. Heat waves were characterized by their intensity, duration, and timing in season. Within each community, we estimated mortality risk during each heat wave compared with non-heat wave days, controlling for potential confounders. We combined individual heat wave effect estimates using Bayesian hierarchical modeling to generate overall effects at the community, regional, and national levels. We estimated how heat wave mortality effects were modified by heat wave characteristics (intensity, duration, timing in season).

**Results:**

Nationally, mortality increased 3.74% [95% posterior interval (PI), 2.29–5.22%] during heat waves compared with non-heat wave days. Heat wave mortality risk increased 2.49% for every 1°F increase in heat wave intensity and 0.38% for every 1-day increase in heat wave duration. Mortality increased 5.04% (95% PI, 3.06–7.06%) during the first heat wave of the summer versus 2.65% (95% PI, 1.14–4.18%) during later heat waves, compared with non-heat wave days. Heat wave mortality impacts and effect modification by heat wave characteristics were more pronounced in the Northeast and Midwest compared with the South.

**Conclusions:**

We found higher mortality risk from heat waves that were more intense or longer, or those occurring earlier in summer. These findings have implications for decision makers and researchers estimating health effects from climate change.

Heat waves can have large impacts on human health; mortality occasionally more than doubles during heat waves (e.g., Oechsli and Buechley 1970; Whitman et al. 1997). Interest in related health effects increased after major heat waves (i.e., Chicago, IL, 1995; Europe, 2003) (Le Tertre et al. 2006; Semenza et al. 1996) and with projections that climate change may increase the frequency, duration, and intensity of heat waves (Meehl and Tebaldi 2004). Understanding how heat waves affect health is key to preparing communities for heat waves and to estimating the health impacts of climate change.

Most studies of mortality and heat estimated health effects during specific heat waves (e.g., Kaiser et al. 2007; Weisskopf et al. 2002) or applied time-series or case-crossover methods to estimate the effects of single days of heat. Few studies combined approaches to consider the effects of both heat waves and single days of high temperature (Anderson and Bell 2009; Hajat et al. 2006); they showed additional health effects from prolonged heat beyond the sum of anticipated effects associated with single hot days. These studies, however, did not distinguish between effects of individual heat waves, but rather estimated health responses assuming that all heat waves of a specified definition have the same impact on health.

The study of heat waves faces several challenges. Heat waves are usually defined as extended periods of extreme heat, although no consistent definition exists regarding the temperature threshold, temperature metric, and number of days used to define heat waves. For example, studies have used thresholds of mean temperature (Hajat et al. 2006), apparent temperature (Smoyer 1998), or combinations of thresholds of apparent and minimum temperature (Robinson 2001; Weisskopf et al. 2002). Use of various heat wave definitions results in different time periods being classified as heat waves, hindering comparison and synthesis of results across studies. Further, heat waves differ in their intensity (degree of heat) and duration. Although most studies use measures of intensity and duration to define a heat wave, few have investigated how these heat wave characteristics affect the mortality impact.

Early studies of one or a few heat waves proposed that differences between the health effects of different heat waves might relate to a heat wave’s intensity, duration, or timing in the summer, although these studies did not investigate effect modification (e.g., Ellis et al. 1975; Schuman 1972). Research of heat waves in New York, New York (Marmor 1975); Madrid, Spain (Diaz et al. 2002); and St. Louis, Missouri (Smoyer 1998) found that mortality effects of heat waves decreased as summer progressed. Studies estimating other elements of the temperature–mortality relationship also found differences in effects with timing in season. Effects of single days of high temperature were larger earlier in the summer in several U.S. and European cities (e.g., Baccini et al. 2008; Hajat et al. 2002; Kalkstein and Smoyer 1993). A study in Philadelphia, Pennsylvania, using a synoptic approach found that oppressive air masses had greater effects when they occurred earlier in the summer (Kalkstein et al. 1996). Of this research, only the single-city studies considered heat waves (i.e., extended periods of high temperature) (Diaz et al. 2002; Marmor 1975; Smoyer 1998).

Other studies provided evidence of the importance of heat wave duration and intensity. One international study showed that duration sometimes modified heat wave mortality effects (Kalkstein and Smoyer 1993). Studies of Madrid (Diaz et al. 2002) and St. Louis (Smoyer 1998) found greater mortality effects for longer heat waves. The duration and intensity of oppressive air masses sometimes modified their mortality effects (Kalkstein and Greene 1996; Kalkstein et al. 1996). Two studies found that average mortality effects increase when heat wave definitions are limited to longer or more intense heat waves (Anderson and Bell 2009; Hajat et al. 2006).

In this study, we estimated the mortality effects of heat waves across the United States (43 communities) for the period 1987–2005 and determined how these effects changed when heat waves were more intense, longer, or earlier in the summer. Although several studies have suggested that heat wave characteristics affect mortality risk, to our knowledge this is the first multicity study of prolonged periods of high temperature (heat waves) in the United States to investigate the impacts of heat wave timing and the largest to examine effect modification by heat wave duration and intensity. This research is also one of the largest studies to date of heat wave effects in the United States. We combined episodic and time-series approaches to estimate the risk of nonaccidental mortality during each individual heat wave compared with risk on non-heat wave days. Then we estimated whether heat wave intensity, duration, or timing in season explained variability in effect estimates. We examined regional differences in heat wave effects and in the modification of effects by heat wave characteristics.

## Materials and Methods

### Data and heat wave selection

Daily nonaccidental mortality data for 108 U.S. urban communities (1987–2005) were obtained from an extended version of the National Morbidity, Mortality, and Air Pollution Study data set (originally 1987–2000) (Anderson and Bell 2009; Internet-Based Health and Air Pollution Surveillance System 2005). Communities were defined as contiguous counties of a metropolitan area (Bell et al. 2004). We omitted data for noncontinental communities, those with population < 200,000 (Dominici et al. 2006; Peng et al. 2008), and those without weather data for ≥ 99.5% of study days, leaving 59 communities.

Weather data came from the National Climatic Data Center (2011). Daily relative humidity was calculated from dew point temperature (Bosen 1958) and used to calculate daily apparent temperature, a metric incorporating air temperature and relative humidity to better approximate the physical experience of heat (Robinson 2001). Dew point temperature is correlated with air temperature and was adjusted for daily mean temperature to limit collinearity in the model (after adjustment, average community correlation between daily dew point temperature and maximum temperature = 0.12) (Bell et al. 2004). We limited analysis to the warm season (1 May–30 September).

A heat wave consists of consecutive days with temperatures above a threshold temperature that can either be physiologically based (absolute threshold) or location based (relative threshold) (Robinson 2001). We used a relative threshold based on the community’s own long-term weather, to allow for regional acclimatization to temperatures normal for a community. We identified heat waves as ≥ 2 consecutive days with daily mean temperature (*T*_mean_) higher than the community’s 95th percentile warm season *T*_mean_ (for 1987–2005), a definition similar to those used previously (Anderson and Bell 2009; Hajat et al. 2006).

Sixteen of the 59 communities had very mild summers (90th percentile summertime mean apparent temperature < 80°F). Under our heat wave definition, the threshold temperature for a heat wave was low in these communities (e.g., 67.3°F in San Francisco, CA). Even though such temperatures are rare for these communities, we did not categorize days with these low temperatures as heat waves. Therefore, we excluded these communities with mild climates. Our final data set included 43 communities [see Supplemental Material, Table 1 (doi:10.1289/ehp.1002313)].

### Identification and characterization of heat waves

We first identified all heat waves in each community for 1987–2005 using the community-specific heat wave definition. We characterized each heat wave by its intensity, duration, and timing in season. Heat wave intensity measured average *T*_mean_ during the heat wave [other metrics for heat wave intensity were considered in sensitivity analysis; see Supplemental Material, Table 2 (doi:10.1289/ehp.1002313)]. Heat wave duration measured the heat wave’s length in days. We characterized the timing of the heat wave in the summer in two ways. First, timing in season measured the day in the season when the heat wave started (with 1 May = 0, 2 May = 1, etc.). Second, first in season identified whether the heat wave was the first heat wave of its year. Supplemental Material, Table 3 (doi:10.1289/ehp.1002313), gives an example of this heat wave classification.

### Association between heat waves and mortality

For each community, we estimated the increase in nonaccidental mortality risk during each heat wave compared with non-heat wave days, controlling for daily temperature. Several studies found that mortality risk increases on individual days of heat (e.g., Anderson and Bell 2009; Baccini et al. 2008). Hajat et al. (2006) discussed the concept of an added heat wave effect and evaluated whether heat wave days affected mortality risk differently than nonconsecutive individual days of high temperatures. This effect has been used to quantify the effects of single notable heat waves [e.g., the 2003 French heat wave (Le Tertre et al. 2006), the 1995 Chicago heat wave (Kaiser et al. 2007)] and to quantify effects of all heat waves over a study period (Hajat et al. 2006; Rocklov and Forsberg 2008).

We estimated this added heat wave effect for each heat wave using community-specific generalized linear models. We controlled for daily maximum temperature to separate effects of heat waves from effects of single days of hot temperature; we also controlled for day of the week and adjusted dew point temperature and time trends to account for seasonal and long-term changes in mortality patterns in a community. We used a nonordered categorical factor to identify heat waves. This variable took a different value for each heat wave. This approach is similar to methods used in earlier studies to compare mortality risk on heat wave days with non-heat wave days (Anderson and Bell 2009; Hajat et al. 2006); however, our model extends the earlier method by allowing risk of mortality to differ by heat wave. Because we estimate a separate effect for each heat wave, we can investigate effect modification by heat wave characteristics (e.g., duration). The model can be stated as


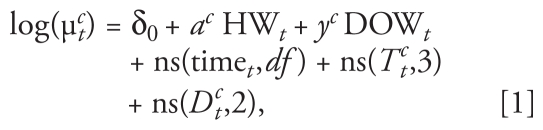


where μ*_t_^c^* = expected mortality rate for community *c* on day *t*; δ_0_ = model intercept; *a^c^* = vector of regression coefficients for heat waves for community *c* (one per heat wave); HW*_t_* *=* 0 if day *t* is a non-heat wave day, 1 if day *t* is the first day of any heat wave, 2 if day *t* is the second or later day in the first heat wave in the community, 3 if day *t* is the second or later day in the second heat wave in the community, and so forth; *y^c^* = vector of regression coefficients for day of the week for community *c*; DOW*_t_* = categorical variable for day of the week; ns(time*_t_*) *=* natural cubic spline of time, with 3 degrees of freedom (df) per warm season (1 May–30 September); ns(*T_t_^c^*) = natural cubic spline of maximum temperature for community *c* for day *t* (df = 3 with knots at quantiles); and (*D_t_^c^*) = natural cubic spline of adjusted dew point temperature for community *c* on day *t* (df = 2).

Equation 1 estimates a separate mortality effect for each heat wave in a community. We used a community-specific Bayesian hierarchical model to generate an overall heat wave effect for each community by combining effects of individual heat waves within that community, incorporating the estimates’ variance (Everson and Morris 2000; Kass and Wasserman 1996):


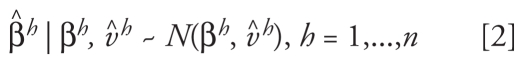



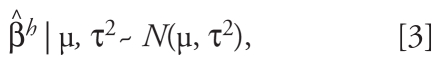


where β̂*^h^* = estimated effect of heat wave *h* on mortality, β*^h^* = true effect of heat wave *h* on mortality, *v̂^h^* = statistical variance of β̂*^h^*, μ = true average heat wave effect across all heat waves, τ^2^ = between-heat wave variance of the true effect, and *n* = number of heat waves.

This model was fit separately for each community. This method is often used to combine effect estimates across communities in air pollution and temperature studies (Anderson and Bell 2009; Bell and Dominici 2008). National and regional heat wave effects were estimated using similar multistage hierarchical Bayesian models. Results were generated separately for three U.S. regions, Northeast, Midwest, and South, based on regions used previously (Anderson and Bell 2009; Barnett 2007). Insufficient numbers of communities precluded estimates for other regions.

### Association between heat wave characteristics and heat wave mortality risk

Within each community, we estimated the relationship between each heat wave characteristic (i.e., intensity, duration, or timing in season) and heat wave effects using a hierarchical Bayesian model, with the heat wave characteristic as an independent variable:


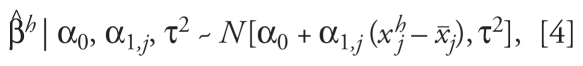


where *x_j_^h^* = heat wave characteristic *j* (intensity, duration, or timing) for heat wave *h*, *x̄_j_* = mean characteristic *j* across all heat waves, α_0_ = average ln(relative rate) when *x_j_^h^* = *x̄_j_*, α_1_*_,j_* = change in ln(relative rate) for unit increase in *x_j_^h^* − *x̄_j_*, and τ^2^ = variance of heat wave effects. We repeated this model separately for each heat wave characteristic and community.

### Sensitivity analysis and extreme events analysis

Use of different heat wave definitions can change the days identified as heat waves [see Supplemental Material, Figure 1 (doi:10.1289/ehp.1002313)]. As a sensitivity analysis, we recalculated main results using different heat wave definitions. Similarly, we investigated effect modification using a variety of metrics for heat wave intensity.

Some heat waves have had alarming health effects. For example, > 3,000 excess deaths occurred in France during a 2003 heat wave (Le Tertre et al. 2006). Previous researchers hypothesized that a heat wave might have a larger effect if it is more intense or longer or occurs at the beginning of summer (Ellis et al. 1975; Schuman 1972). We investigated whether these characteristics could explain the catastrophic health effects of certain heat waves.

We defined a catastrophic heat wave as one associated with a mortality effect estimate at least three times larger than that of any other heat wave in the same community during our study period. Only a few communities in our study experienced a catastrophic heat wave during our study period. (However, this does not mean that the other communities cannot or will not experience similarly devastating heat waves.) For each community that experienced a catastrophic heat wave, we created a multivariate Bayesian hierarchical model for heat wave effects using 18 years of data (all years except the year of the catastrophic heat wave) with terms for heat wave intensity, duration, and timing in season. We then estimated the mortality effect for the catastrophic heat wave based on this model and compared results with the actual mortality effect.

## Results

### Characteristics of heat waves

**Figure 1:**
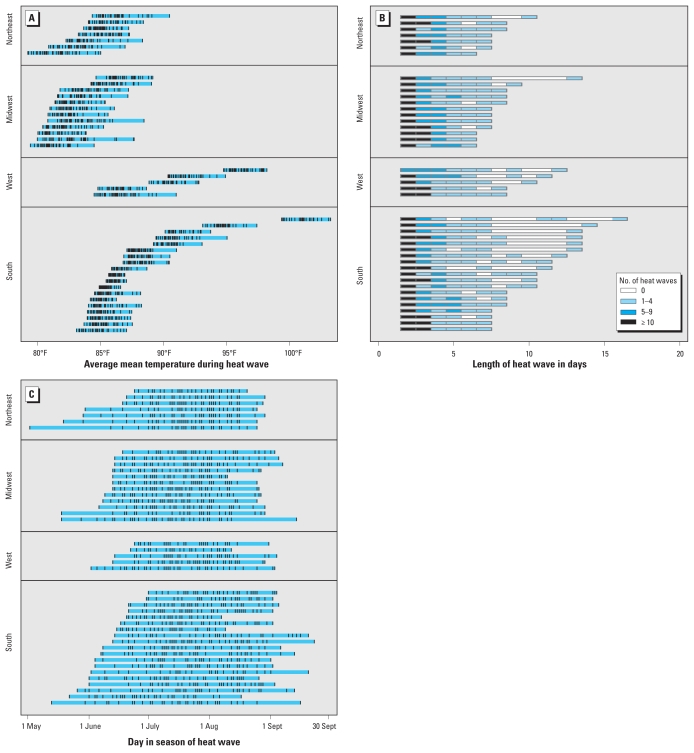
Heat wave characteristics in 43 individual communities (1987–2005). Each horizontal line represents one community, and the length of each line indicates the range of each heat wave characteristic in that community. (*A*) Intensity of each individual heat wave within a community (tick marks within blue bars). (*B*) Number of heat waves according to duration (shading) by community. By definition, all heat waves lasted ≥ 2 days. (*C*) Heat wave timing in season by community (tick marks, individual heat waves).

Supplemental Material, Figure 2 (doi:10.1289/ehp.1002313), maps the communities included in this study. Under our heat wave definition (≥ 2 consecutive days with daily mean temperature, *T*_mean_, higher than the community’s 95th percentile summertime *T*_mean_), communities in the study experienced on average 1.9 heat waves/year, with little variation by region (Table 1). Heat waves varied across regions in intensity, duration, and timing in season (Figure 1). Longer and more intense heat waves were more common in the U.S. South, although heat wave characteristics showed wide ranges within each community (Figure 1, Table 1). Most identified heat waves were 2–3 days; heat waves > 7 days were rare in the North and Midwest, and heat waves > 10 days were rare in any community (Figure 1B). Early or late heat waves (i.e., in May or September) were rare (Figure 1C). In most communities, more intense heat waves were also likely to be longer and earlier in the season, although the three heat wave characteristics were not highly correlated [see Supplemental Material, Table 4 (doi:10.1289/ehp.1002313)].

### Association between heat waves and mortality

We found, on average, higher risk of mortality during heat waves than during non-heat wave periods, with variation in health effects of different heat waves (Table 2). Effect estimates were much lower in the South than in the Northeast or Midwest (Table 2), although the threshold temperatures used to define heat waves (95th percentile of warm season daily *T*_mean_) were higher in the South [see Supplemental Material, Figure 2 (doi:10.1289/ehp.1002313)]. Some southern cities (e.g., Charlotte, NC; Dallas/Fort Worth, TX; Oklahoma City, OK; Tulsa, OK) showed no mortality increase during heat waves.

### Impact of heat wave characteristics on mortality risk

**Figure 2:**
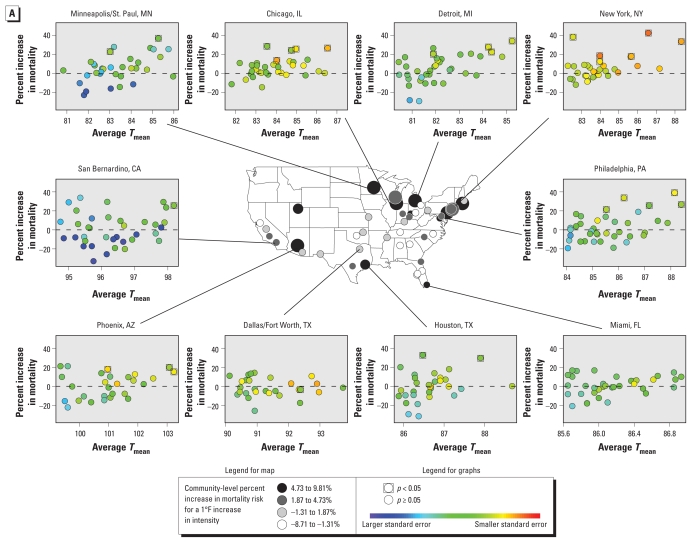

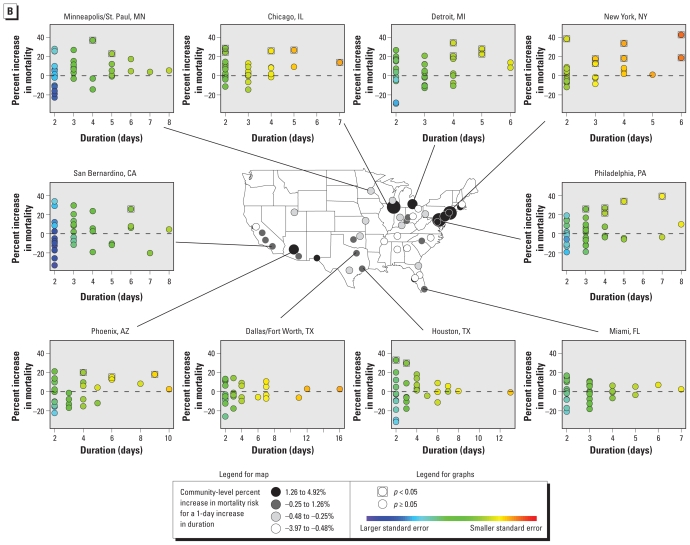

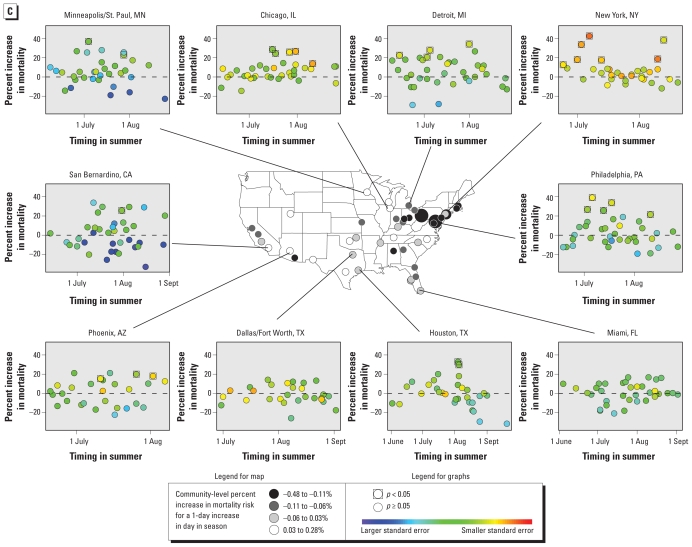
Heat wave characteristics and heat wave effects in 43 communities (1987–2005): associations between heat wave effects (percent mortality increase during the heat wave vs. non-heat wave days) and average *T*_mean_ during each heat wave (*A*), heat wave duration (*B*), and timing in season of the heat wave (*C*). Maps show relative risks of nonaccidental mortality during heat wave days compared with non-heat wave days (controlling for daily temperature) and heat wave characteristics. On the map, the color of each community’s circle reflects the magnitude of effect modification; the size reflects the statistical variance, with larger circles indicating more precise estimates. Graph insets plot the value of each heat wave characteristic versus its nonaccidental mortality effect [estimated as an added heat wave effect (Hajat et al. 2006)] for the 10 most populous communities. For these graphs, each circle represents an individual heat wave, and the circle’s color reflects the variance of the mortality effect. The July 1995 Chicago heat wave and August 1988 Minneapolis/St. Paul heat wave are omitted as they are outside the range of the figures. Scales on *x*-axes differ among the graphs for individual cities.

The health effects of individual heat waves were associated with heat wave intensity, duration, and timing in many of the communities, with large heterogeneity across communities. Figure 2 shows community-specific plots of heat wave effects versus heat wave characteristics for the 10 most populous communities and maps of the overall association in each community. In general, all three heat wave characteristics were more strongly associated with heat wave effects in the Northeast and Midwest than in the South (Figure 2, Table 3). A 1°F increase in average *T*_mean_ during a heat wave was associated with a 4.39% increase in the relative risk of mortality during that heat wave in the Northeast and a 3.22% increase in the Midwest. The association between heat wave duration and mortality effects was largest in the Northeast, where mortality risk during a heat wave was on average 2.50% higher for every extra day a heat wave lasted.

We divided heat waves into two groups: those that were the first to occur in a year (first in season) and those that were preceded by another heat wave in their year (not first in season) for a given year and community. On average, we categorized 40% of all heat waves as first in season. First-in-season heat waves generally had higher effects than did later heat waves, although effects were similar in the Midwest (Table 4). The importance of timing in the season on heat wave effects was also evident when we considered the influence of day in season (i.e., which day of the season the heat wave started) on heat wave effects, particularly in the Northeast (Figure 2C, Table 3).

### Sensitivity analysis

We reestimated mortality risk during heat waves compared with non-heat wave days using different heat wave definitions [see Supplemental Material, Tables 2 and 5 (doi:10.1289/ehp.1002313)]. Point estimates changed but trends were similar under all definitions considered: Average heat wave effects were positive and significant, more intense and longer heat waves were positively associated with heat wave effects, and the first heat waves in the summer almost always had a higher average effect than later heat waves. Heat wave intensity was significantly associated with heat wave effects for almost all heat wave definitions [see Supplemental Material, Table 5 (doi:10.1289/ehp.1002313)]. Regional trends were largely consistent regardless of heat wave definition, with higher, more significant heat wave effects in the Northeast than in other areas and a stronger association between heat wave intensity and heat wave effects in the Northeast (data not shown).

We tested effect modification by heat wave intensity using several different metrics for intensity [see Supplemental Material, Tables 2 and 7 (doi:10.1289/ehp.1002313)]. For each temperature metric, we considered the effect of the peak value (the highest value of that metric during a single day of the heat wave) and the average value across all days of the heat wave. Metrics of heat wave intensity were not all strongly correlated across communities [see Supplemental Material, Table 6 (doi:10.1289/ehp.1002313)], although intensity measures based on the peak value of a particular temperature metric and the average value of the same temperature metric were highly correlated (0.87–0.93). Supplemental Material, Table 7 (doi:10.1289/ehp.1002313), shows the percent increase in mortality risk for heat wave days compared with non-heat wave days for a 1°F increase in heat wave intensity, under different heat wave intensity definitions. More intense (i.e., higher temperature) heat waves were associated with higher mortality risk under all definitions of heat wave intensity.

### Analysis of extreme heat waves

In a separate analysis, we analyzed effects of particularly extreme heat waves. We identified two catastrophic heat waves; both involved the same weather system: 12–16 July 1995 in Chicago and 13–15 July 1995 in Milwaukee, Wisconsin. Both heat waves were the most intense in their community over the study period (average *T*_mean_ = 87.2°F for Chicago, 87.7°F for Milwaukee) [see Supplemental Material, Figure 3 (doi:10.1289/ehp.1002313)]. They were not extreme in duration or timing in the summer (both were in July, and they lasted 5 and 3 days, respectively). Mortality risks during these heat waves, compared with non-heat wave days (using Equation 1), were 133.9% [95% posterior interval (PI), 116.9–152.2%] for Chicago and 93.0% (95% PI, 53.1–143.3%) for Milwaukee.

We investigated whether the mortality risk of these two events could be explained by their intensity, duration, and timing in season. We fitted multivariate community-specific models separately for Chicago and Milwaukee, excluding heat waves during the year of the catastrophic heat wave (1995). We then used these two models to estimate the expected mortality risk from the two catastrophic heat waves and found an increase in mortality risk of 18.9% for the Chicago catastrophic heat wave and 10.3% for Milwaukee, much smaller than the mortality risks observed.

## Discussion

### Within-community heterogeneity in heat wave effects

Estimated overall heat wave effects were similar to those calculated in an earlier study of U.S. heat waves that included more communities over a shorter time period (1987–2000); however, that previous study did not investigate variation in effects of individual heat waves within a community or explore how heat wave characteristics modify mortality risk (Anderson and Bell 2009). We found that, within a community, heat wave mortality effects are influenced by the heat wave’s intensity, duration, and timing in the season. This effect modification probably results from physiological responses to heat and/or behavior modification.

When ambient temperature is high, the human body responds via thermoregulation: Blood vessels dilate near the skin to transfer heat from the body’s core to the skin, and then sweat transfers heat from the skin by evaporation (Havenith 2005). Even when body temperature remains normal, thermoregulation strains the cardiovascular system (Havenith 2005). The higher the temperature or the longer the heat wave, the more work required of the cardiovascular system to maintain normal temperature; therefore, more intense or longer heat waves are likely to have greater health effects.

Heat wave timing in the season might influence mortality risk from *a*) deaths of particularly susceptible individuals during heat waves or single hot days earlier in the summer, leaving a smaller pool of susceptible individuals later in the summer (mortality displacement), or *b*) acclimatization of individuals to heat over the course of a summer. Evidence of both effects exists: studies considering health effects of both single days of heat (Baccini et al. 2008; Braga et al. 2001; Hajat et al. 2005) and of specific heat waves (Kaiser et al. 2007; Le Tertre et al. 2006) found evidence of mortality displacement, whereas experimental studies found that humans acclimatize to heat, usually within several weeks of exposure (Bouchama and Knochel 2002). We found that heat waves earlier in summer had higher mortality risk than did later heat waves and that the first heat wave of the summer had a higher impact than did later heat waves. Additional research is needed to determine whether the lessening impact of heat waves over the summer is due to mortality displacement, biological adaptation (e.g., persons becoming physically accustomed to higher temperatures), and/or behavior modification [e.g., increased use of air conditioning (AC), staying indoors]. Additional research could also investigate if the timing of heat waves has a nonlinear relationship with mortality effects.

The characteristics of heat waves considered here (intensity, duration, and timing in season) could not fully explain variation in heat wave effects; for example, often heat waves of the same intensity or length affected mortality very differently within a community. Other possible sources of variation in heat wave mortality risk within a community include changes over time in communities’ responses to heat waves (e.g., implementation of heat wave warning systems) and changes in public perception of heat waves (e.g., willingness to take protective measures) (Kalkstein et al. 1996; Kovats and Ebi 2006). Power outages sometimes occur during heat waves (e.g., Bridger et al. 1976; Luber and McGeehin 2008), and these could contribute to particularly high impacts due to increased heat exposure without AC and greater difficulty leaving upper-level apartments in buildings with elevators. Often studies cite the differences between the effects of two heat waves as evidence of improvements in community response (e.g., Palecki et al. 2001; Weisskopf et al. 2002). This study shows a large variation in heat wave effects for a variety of reasons, suggesting that studies analyzing the efficacy of response measures should not rely on differences between only two heat waves or ignore differences in heat wave characteristics.

### Between-community heterogeneity in heat wave effects and effect modification

Heterogeneity between communities in both heat wave effects and effect modification by heat wave characteristics might result from *a*) physical acclimatization of residents of warmer communities, *b*) different levels of exposure in different communities (e.g., AC use, housing structure, clothing type), *c*) different community-level responses to extreme heat (e.g., heat wave warning systems), *d*) different demographics (e.g., population in high risk categories such as the elderly or less healthy), or *e*) different geographical, meteorological, or pollution factors within communities that might confound or modify temperature–mortality relationships.

We observed general regional patterns in community-level heat wave effects that are consistent with other studies of heat waves or high temperature, which report highest effects in the U.S. Northeast and Midwest (e.g., Anderson and Bell 2009; Braga et al. 2001). Some researchers found that temperature effects were related to AC (e.g., Braga et al. 2001; O’Neill et al. 2005), although an early study noted similar geographic patterns before widespread AC prevalence (Gover 1938). In contrast, patterns are reversed in Europe, where communities with warmer climates generally had larger heat effects (Baccini et al. 2008).

Heterogeneity in effect modification by heat wave characteristics followed similar regional patterns. Heat wave intensity, duration, and timing in the summer had smaller effects on mortality in the South than elsewhere. Differences between the South and other parts of the country in exposure to outdoor air temperature (e.g., because of AC use) might explain why even very long and intense heat waves failed to affect mortality in some communities.

### Sensitivity analysis

Different studies have used a variety of definitions to select what days constitute “heat waves.” Additionally, a variety of theories exist concerning which temperature metric should be used to best capture the heat wave periods most dangerous to human health. Some researchers propose that high minimum temperatures might be particularly important, theorizing that the body is more affected with no relief from cool nighttime temperatures (Schwartz 2005). Others suggest the use of apparent temperature, because the body may be more stressed if humidity limits cooling by sweat evaporation (Robinson 2001). This question is complex, and the appropriate metric may vary by individual, depending on health status and exposure. For example, minimum temperature might be important for someone without AC but a less valuable metric for someone living in a home with central AC. We performed sensitivity analysis under a variety of heat wave definitions (intensity, duration, metric) to aid interpretation and meaningful comparison with other studies.

We did not control for air pollution in our model, because earlier research showed that temperature effects in the United States were robust to air pollution (Anderson and Bell 2009). However, air pollution and temperature might act synergistically during heat waves; future research could investigate this question.

### Extreme heat waves

Several days early in July 1995 qualified as a heat wave in many of the Midwest and Northeast communities in our study; mortality effects were particularly extreme in Chicago and Milwaukee. The July 1995 heat wave was unusual in this data set in terms of the magnitude of mortality effects, although it was not particularly long or early in the season. We found that the extremity of this heat wave’s effects could partially be explained by its intensity, although comparison is difficult because the heat wave is outside the range of other heat waves in terms of intensity and mortality. Although we found heat wave mortality risk to be affected by intensity, duration, and timing in season, these factors could not fully explain catastrophic heat wave effects. Some variation may relate to changes from summer to summer in the number of people in a community who are very susceptible to heat. For example, one study found that heat-related mortality can be affected by rates of influenza-related mortality the preceding winter (Rocklov et al. 2009). Other factors, possibly social, may also play a role. Such factors, including changes in community and public health responses to heat, are beyond the scope of this study and could be explored in future research. The finding that catastrophic heat wave effects cannot be adequately explained by heat wave intensity, duration, and timing in the summer highlights the difficulty of predicting catastrophic heat waves, either with heat warning systems or with long-term climate change projections.

A meteorologic study showed that heat waves of similar intensity to the July 1995 heat wave are very rare in Chicago, although not unprecedented in the 20th century (Kunkel et al. 1996). Earlier studies of individual heat waves in the United States used different methods to estimate excess mortality but showed that mortality effects as high as those observed in Chicago and Milwaukee in July 1995 have occurred. Estimated approximate mortality increases during other extreme heat wave periods, compared with non-heat wave periods, for Los Angeles, California, are 109% in late September 1939 and 112% in early September 1955 (Oechsli and Buechley 1970); for St. Louis, Missouri, are 91% in July 1966 (Bridger et al. 1976) and 159% in 9–14 July 1966 (Henschel et al. 1969); and for New York, New York, are 113% in 28 August through 3 September 1948 (Ellis and Nelson 1978).

## Conclusions

Study of heat waves’ effects on human health is important for the present day and central to estimating the impacts of climate change. Heat waves are anticipated to be more common, longer, and more intense in the future (Meehl and Tebaldi 2004). This study is unique among heat wave studies in its national scale, long time frame, multiple heat wave definitions, and consideration of how heat wave mortality effects are associated with heat wave intensity, duration, and timing in the season. We found that mortality risk increased during prolonged periods of extreme heat, compared with the community’s usual climate, and estimated how this mortality risk was affected by heat wave intensity, duration, or timing within the year. Because of the national scale, we were able to examine regional trends and observed both larger heat wave effects and greater effect modification by heat wave characteristics in certain regions, particularly the Northeast; trends were less defined or in some cases inversed in some communities in the South. Heat wave–related mortality under a changing climate will need to address acclimatization over a long time frame (e.g., changes over years) and a shorter time frame (e.g., changes within a summer). Additionally, the observed heterogeneity, both in heat wave effects and in the influence of heat wave characteristics on mortality effects between different communities, indicates the importance of developing heat wave response plans that are community specific.

## Figures and Tables

**Table 1:** Summaries of heat wave characteristics (1987–2005).

	Heat wave characteristic			
Region	No./year/community	Intensity (°F)	Duration (days)	Day in season
National (*n* = 43)	1.9	86.4	3.3	21 July
Northeast (*n* = 7)	1.9	84.4	3.1	21 July
Midwest (*n* = 12)	1.9	83.3	3.2	18 July
South (*n* = 19)	1.8	88.1	3.4	23 July

For each characteristic shown, community-specific averages were calculated and then averaged nationally or by region.

**Table 2:** Summary of heat wave mortality effects (1987–2005).

Region	Heat wave effect (95% PI)
National (*n* = 43)	3.74% (2.29 to 5.22%)
Northeast (*n* = 7)	6.76% (1.79 to 11.98%)
Midwest (*n* = 12)	5.62% (3.36 to 7.93%)
South (*n* = 19)	1.84% (−0.11% to 3.84%)

The heat wave effect is the increase in nonaccidental mortality risk for heat wave days compared with non-heat wave days, controlling for daily temperature (the added heat wave effect described by Hajat et al. 2006).

**Table 3:** Percent increase in relative risk of mortality during a heat wave per unit increase in heat wave characteristic (1987–2005).

Region	Increase of 1°F in average *T*_mean_	One-day increase in duration	One-day increase in timing in season (1 May = 1)
National (*n* = 43)	2.49%***	0.38%	−0.063%**
Northeast (*n* = 7)	4.39%***	2.50%**	−0.227%**
Midwest (*n* = 12)	3.22%***	0.09%	−0.071%
South (*n* = 19)	0.43%	0.08%	−0.022%

Data are percent increase in the relative risk of nonaccidental mortality on heat wave days compared with non-heat wave days, controlling for daily temperature, for a unit increase in the heat wave characteristic.

** *p* < 0.05;

*** *p* < 0.01.

**Table 4:** Average mortality effects of the first heat wave in a summer versus later heat waves (1987–2005).

Region	Average percentage of heat waves that were first in season	Average effect of heat waves (95% PI)	
		First in season	Not first in season
National (*n* = 43)	40%	5.04% (3.06 to 7.06%)	2.65% (1.14 to 4.18%)
Northeast (*n* = 7)	40%	11.08% (4.05 to 18.58%)	3.45% (−1.16 to 8.28%)
Midwest (*n* = 12)	38%	5.29% (1.76 to 8.94%)	5.42% (2.46 to 8.46%)
South (*n* = 19)	38%	3.29% (0.12 to 6.56%)	0.68% (−1.60 to 3.02%)

The heat wave effect is the increase in nonaccidental mortality risk for heat wave days compared with non-heat wave days, controlling for daily temperature [the added heat wave effect described by Hajat et al. (2006)].
